# Analysis of the Cytokine Expression in the Aqueous Humor of Individuals with BRVO-Associated Macular Edema

**DOI:** 10.1155/2022/1514244

**Published:** 2022-08-04

**Authors:** Chengyi Zhu, Lanlan Pan, Qiong Yi, Qiping Wei

**Affiliations:** ^1^Ophthalmology Department, Beijing He Ping Li Hospital, Beijing 100013, China; ^2^Ophthalmology Department, Beijing Oriental Hospital, University of Traditional Chinese Medicine, Beijing 100078, China

## Abstract

**Purpose:**

This study aimed to determine the expression levels of vascular endothelial growth factor (VEGF), interleukin-6 (IL-6), intercellular adhesion molecule-1 (ICAM-1), and vascular cell adhesion molecule-1 (VCAM-1) in the aqueous humor of patients with macular edema (ME) caused by branch retinal vein occlusion (BRVO), as well as to investigate the relationship between the cytokines as mentioned earlier and best-corrected visual acuity (BCVA), ME, and the degree of ME from the molecular level.

**Methods:**

In a prospective observational study, fluorescein fundus angiography (FFA) and optical coherence tomography (OCT) were used to classify 58 patients with non-ischemic BRVO-ME into three groups according to the degree of ME: 14-mild, 17-moderate, and 27-severe. The specific concentration of IL-6, VEGF, ICAM-1, and VCAM-1 in the aqueous humor was detected using the BD CSCanto™ II Flow Cytometer (US). Spearman or Pearson correlation analysis was used to test the correlation between the levels of BCVA and severity of ME and the expression levels of IL-6, VEGF, ICAM-1, and VCAM-1 in the aqueous humor.

**Results:**

According to the obtained data, BCVA did not correlate with the severity of ME, and these four cytokines expression levels in patients' aqueous humor (*P* > 0.05). Moreover, BCVA did not correlate with mild, moderate, or severe ME as well (*P* > 0.05). However, the levels of these four cytokines were correlated with the severity of the ME. These underlined cytokines were linked to the mild, moderate, and severe degrees of ME. VEGF was also significantly correlated (*r* > 0.8, *P* < 0.0001) with the severity of ME.

**Conclusions:**

This study suggests that the severity of ME in BRVO-ME patients is significantly correlated with the expression levels of IL-6, VEGF, ICAM-1, and VCAM-1 in the aqueous humor. Lowering the level of disease-associated cytokines may potentially reduce the degree of ME. Therefore, an in-depth study of the levels and the relationship may provide some evidence for the pathogenesis, treatment, and prevention of BRVO-ME.

## 1. Introduction

Branch retinal vein occlusion (BRVO) is a common fundus vascular disease. According to a 2015 epidemiological survey, the prevalence of BRVO among adults aged 30–89 years was 0.64% worldwide, with cumulative 5 and 10 years rates of 0.86% and 1.63%, respectively, with Asians having the highest prevalence rate. The estimated number of patients is 23.38 million worldwide, most of them over 50 years of age [1, 2]. The macular edema (ME) caused by BRVO is the main cause of visual impairment and even blindness, with an incidence rate of about 30% [3–6].

After the occurrence of BRVO, the venous blood flow in the blocked area is stagnant, increasing the pressure in the capillary system, destroying the blood-retinal barrier (BRB), damaging the tight junctions of capillary endothelial cells, and leading to the disruption of the dynamic balance of fluid infiltration and excretion in the macular tissue which results in macular edema [7–9]. First, due to the stagnation of blood flow in the obstructed area, the retina is in a state of ischemia and hypoxia, resulting in strongly expressed VEGF levels. VEGF can activate the VEGFR-1 signaling pathway. Under hypoxia, activated VEGFR-1 can stimulate monocytes/macrophages to strongly express IL-6 and other inflammatory cytokines. By promoting actin filament rearrangement, IL-6 can induce the formation of gap junctions between adjacent cells, thereby increasing the permeability of the vascular endothelium and aggravating the degree of ME [8–16]. Second, VEGF can also activate the VEGFR-2 signaling pathway to increase vascular permeability. Moreover, it can upregulate the expression level of downstream ICAM-1, VCAM-1, and other cytokines through the nuclear factor Kappa beta (NF-*κ*B) signaling pathway. These cytokines can recruit leukocytes for chemotaxis and adhere to the vascular endothelium, leading to leukocyte stasis and even blood flow stagnation, further aggravating the degree of retinal occlusion and expanding the scope of occlusion, aggravating the ischemia and hypoxia of the retina in the occluded area. Additionally, the adhesion of leukocytes to the vascular endothelium can destroy the function of the vascular endothelium and increase the vascular endothelial function. Permeability increases the degree of ME and forms a positive feedback loop [8, 9, 13]. Simultaneously, aberrant leukocyte adhesion results in leukocyte stasis and the blood flow slows, resulting in blood flow stagnation. Additionally, the increased chemotaxis and aberrant adherence of leukocytes will increase the expression of proinflammatory cytokines, enhancing the inflammatory response and establishing another positive feedback loop. [8, 9, 13]. The abovementioned multiple cytokines and signaling pathways are all involved in the formation and development of the BRVO-ME, hypoxia, and inflammation cascades and form a complex network of signaling pathways through multiple positive feedback loops, eventually leading to chronic, recurrent, and refractory ME [4, 14, 17, 18].

Taken together, BRVO-ME is the result of the joint action of a variety of cytokines and their signaling pathways. VEGF, IL-6, ICAM-1, and VCAM-1 are important in the positive feedback loop. However, the relationship between these cytokines and the severity of ME is unclear. Therefore, this study aimed to evaluate the relationship between the severity of ME in BRVO-ME patients and the expression levels of VEGF, IL-6, ICAM-1, and VCAM-1 in the aqueous humor.

## 2. Materials and Methods

### 2.1. Case Selection

#### 2.1.1. Inclusion Criteria

The inclusion criteria were as follows: (1) The diagnosis confirmed by fluorescein fundus angiography [19, 20] (FFA), optical coherence tomography (OCT; Spectralis OCTHeidelberg Engineering, Heidelberg, Germany), non-ischemic BRVO-ME, first onset, and without any treatment; (2) no posterior vitreous detachment found by OCT; (3) those with the diseases of hypertension, diabetes, hyperlipidemia, etc., but no fundus manifestations of related diseases were found on examination of the affected eye and the contralateral eye; and (4) the duration of the disease was less than 1 m.

#### 2.1.2. Exclusion Criteria

The exclusion criteria were as follows: (1) Eye surgery within 3 m; (2) ME caused by diseases other than BRVO-ME; (3) during pregnancy or breastfeeding; (4) a history of acute cardiovascular and cerebrovascular disease within 6 m; (5) history of systemic or local use of hormones within 3 m; and (6) those with severe hepatic and renal insufficiency.

### 2.2. Clinical Data

This prospective observational study was conducted on 58 patients (58 eyes) with non-ischemic BRVO-ME. Each patient received intravitreal ranibizumab injection (IRI) from June 2018 to June 2021 in Beijing Hepingli Hospital. The degree of ME was classified into three categories based on the findings of an OCT examination [21]: mild: 200 *μ*·m ≤ CMT<350 *μ*·m, moderate: 350 ≤ CMT < 500 *μ*·m, and severe: CMT ≥ 500 *μ*·m.

### 2.3. Methods

Each patient received surgery in an aseptic ophthalmic operating room. First, 0.1 ml of the aqueous humor was extracted and kept at −80°C concurrently. Then, IRI was performed. The human IL-6, VEGF, ICAM, and VCAM Plex Flex Set Kit (BD Biosciences) were used in the ELISA procedure. Each index specific concentration was determined using the FACSCanto II flow cytometer (BD Biosciences). The same ophthalmologist collected IRI and aqueous humor samples. Both the IRI and the collection of aqueous humor samples were conducted in compliance with the State Council of the People's Republic of China's “Regulations on Hospital Management.” Each patient signed an informed consent form. The study was authorized and approved under the ethical review number 2018–06.

### 2.4. Statistical Analysis

SPSS 26.0 software was used for descriptive and statistical analysis in this study. Measurement data conforming to the normal distribution were expressed as (*x* *±* *s*) using a *t-*test. Non-conformity to the normal distribution was expressed as M (*P*_*25*_, *P*_*75*_) using the Kruskal–Wallis test. Spearman or Pearson correlation analysis was used to determine correlations, with *r* > 0.5 suggesting a strong correlation, 0.5 > *r* > 0.3 indicating a normal correlation, and *r* < 0.3 indicating a weak correlation. *P* < 0.05 was considered a statistically significant difference.

## 3. Results

### 3.1. Basic Information about Patients

The clinical and demographic characteristics of the 58 patients (58 eyes) are summarized in Table 1.

### 3.2. The Relationship between the Level of BCVA, CMT, VEGF, IL-6, ICAM-1, VCAM-1, and the Severity of ME in the Aqueous Humor

CMT is categorized ME into three degrees based on OCT findings: mild, moderate, and severe. The expression levels of VEGF, IL-6, ICAM-1, and VCAM-1 in aqueous humor varied with the mild, moderate, and severe degrees of ME. However, BCVA did not tend to change with the mild, moderate, and severe degrees of ME (Table 2).

### 3.3. Correlation Analysis of BCVA and the Severity of ME

The Pearson correlation analysis was performed to evaluate the relationship between BCVA and the severity of ME. The obtained data revealed no significant correlation between BCVA and the severity of ME, i.e., mild, moderate, and severe (*P* > 0.05) as shown in Table 3.

### 3.4. Relationship between BCVA and the Expression Levels of VEGF, IL-6, ICAM-1, and VCAM-1 in the Aqueous Humor

The association between BCVA and the expression levels of VEGF, IL-6, ICAM-1, and VCAM-1 in the aqueous humor was investigated using the Pearson correlation analysis. As a result, no significant correlation was observed between BCVA and expression levels of VEGF, IL-6, ICAM-1, and VCAM-1 in the aqueous humor. (*P* > 0.05) as presented in Table 4.

### 3.5. Relationship between the Severity of ME and the Expression Levels of VEGF, IL-6, ICAM-1, and VCAM-1 in the Aqueous Humor

**Figure 1:**
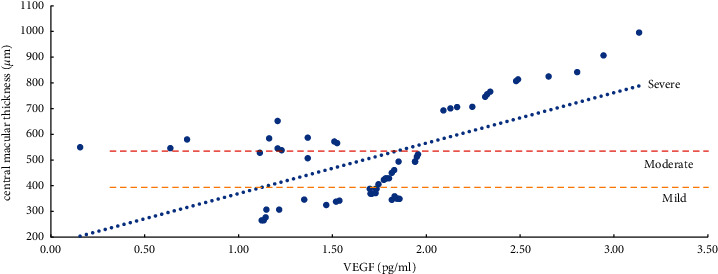
Correlation analysis of ME with a mild, moderate, and severe degree and VEGF (vascular endothelial growth factor).

**Figure 2:**
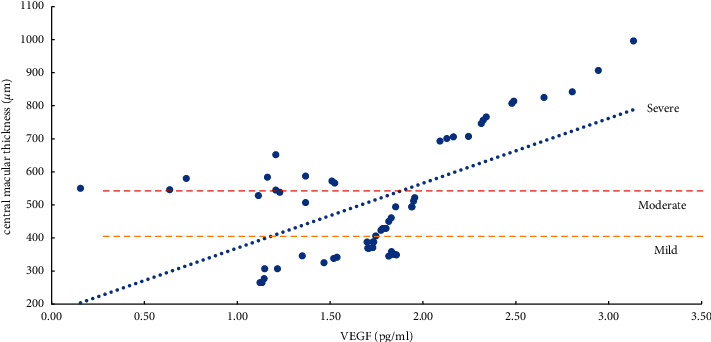
Correlation analysis of ME with a mild, moderate, and severe degree and IL-6 (interleukin-6).

**Figure 3:**
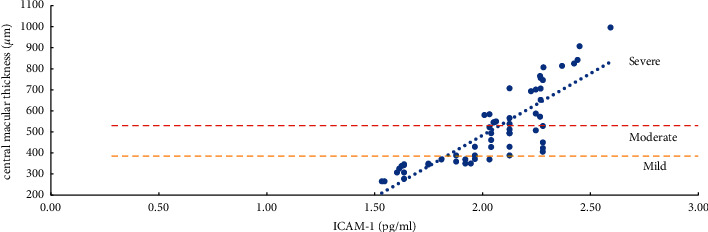
Correlation analysis of ME with a mild, moderate, and severe degree and ICAM-1 (intercellular adhesion molecule-1).

**Figure 4:**
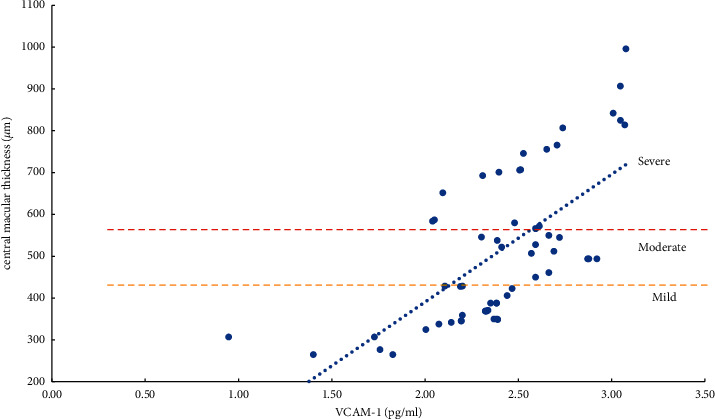
Correlation analysis of ME with a mild, moderate, and severe degree and VCAM-1 (vascular cell adhesion molecule-1).

The severity of ME and the expression levels of VEGF, IL-6, ICAM-1, and VCAM-1 in the aqueous humor were studied using the Pearson correlation analysis. According to the findings, the degree of ME (mild, moderate, and severe) was positively related to VEGF, IL-6, ICAM-1, and VCAM-1 expression levels in the aqueous humor. Except for a moderate correlation between severe ME and VCAM-1 (*r* = 0.4847, *P*=0.0104), the other degrees of ME were found to be strongly correlated (*r* > 0.5, *P* < 0.01) with the level of cytokines (VEGF, IL-6, and ICAM-1) and the mild, moderate, and severe degrees of ME were also positively correlated with the level of VEGF (*r* > 0.8, *P* < 0.0001) (Table 5, Figures 1–4).

## 4. Discussion

BRVO is a highly frequent vascular disorder of the retina found in people with lifestyle-related conditions such as hypertension and arteriosclerosis. Several therapeutic techniques, including laser photocoagulation, have been attempted because the ME is the primary cause of visual impairment in BRVO, but no positive outcomes have been obtained. However, the development of treatments targeting VEGF transformed the therapy of ME. The ME significantly improves with anti-VEGF injection, indicating that VEGF plays a critical role in this disease related to BRVO [7–10]. In this study, a significantly positive correlation was observed between the severity of ME with the expression levels of VEGF, IL-6, ICAM-1, and VCAM-1 in the aqueous humor. Additionally, the percentages of patients with hypertension, diabetes, and hyperlipidemia were 53.45%, 34.48%, and 13.79%, respectively, which are all greater than the figures reported by O'Mahoney et al. [22]. The significant number of these patients in our study was attributable to their advanced age, ranging from 53 to 89 (mean age = 67.46 ± 8.35) years. Jialiang Zhao et al. [23] found that 99% of BRVO patients could find hardened arterioles on the surface of the venule at the obstructed site. In this study, the blockage of BRVO accounts for 65.52% in the supratemporal area and 34.48% in the infratemporal area. The obstruction was not found in the nasal side, which showed consistency with the proportion reported by Nakano E and Samara WA [24, 25].

According to the thickness of CMT, the severity of edema has been divided into three levels: mild, moderate, and severe. Correlations were evaluated using the Spearman or Pearson correlation analysis. Based on the data obtained (*P* > 0.05), no significant association was identified between BCVA and the degree of ME in patients. However, Ryu et al. [26] demonstrated a strong correlation between baseline BCVA and CMT, in contrast to our findings. The finding of our study indicates that the degree of BCVA and ME are not significantly associated, which is similar to the findings of Fujino et al. [27]. This may be because ME traction compromises the integrity of the ellipsoidal zone or that various pathological substances produced during ME formation and development accumulate in the macular area throughout the disease, impairing the ellipsoidal zone's function and the patient's BCVA being correspondingly low. Since long-term edema causes structural damage, the duration of the edema may affect visual function. However, if the ME is restricted to the outer plexiform and inner nuclear layers without affecting the ellipsoid zone, the patient's BCVA will often remain stable. Thus, the change in BCVA has no association with the change in CMT thickness but is directly tied to the ellipsoid belt's function and integrity [27, 28].

BCVA was observed to have no significant correlation with VEGF, IL-6, ICAM-1, or VCAM-1 expression levels in the aqueous humor (*P* > 0.05). This could be because the level of cytokines expressed in aqueous humor does not correctly reflect the real state of the retina, as the level of cytokines expressed in the aqueous humor is modified by their diffusion rate and interaction with the extracellular matrix. The damage of cytokines and their cascades to photoreceptors and neurons in the inner layer of the retina cannot be synchronized [26]. Therefore, there is no apparent correlation between BCVA and the expression levels of VEGF, IL-6, ICAM-1, and VCAM-1 in the aqueous humor.

Herein, the expression levels of VEGF, IL-6, ICAM-1, and VCAM-1 in the aqueous humor varied with the mild, moderate, and severe degrees of ME. The Pearson correlation analysis demonstrated a significantly positive linkage between mild, moderate, and severe ME degrees and the VEGF, IL-6, ICAM-1, and VCAM-1 expression levels in the aqueous humor (*P* < 0.05).

The present study revealed a significantly positive link between VEGF and the degree of mild, moderate, and severe ME, and the correlation coefficients were all above 0.8 (*P* < 0.0001). The reason is that retinal ischemia and hypoxia in the occlusion area after BRVO promotes the strong expression of VEGF, which specifically binds to its receptors (VEGFR-1, VEGFR-2), increases vascular permeability, and upregulates the expression levels of VEGF, IL-6, ICAM-1, VCAM-1, and other inflammatory cytokines, leading to hypoxia-induced inflammation, and further aggravates the degree of ME through multiple positive feedback loops. Chronic, persistent, and refractory ME is caused by a cascade of hypoxia and inflammatory reactions [8]. Thus, VEGF is the primary initiating and mediating factor in the onset and progression of ME [8, 27–31]. Additionally, the reported studies have shown that decreasing VEGF expression can disrupt the downstream positive feedback loop, considerably reduce CMT, and enhance the visual function of BRVO-ME patients [8, 13, 16].

The current study used the same detection methods and reagents as reported by Noma et al. [8, 13] to detect the VEGF value. According to the findings of Noma et al. [8, 13], the VEGF value of the vitreous humor in typical cases is 15.6 pg/ml in mild ischemia, 30–40 pg/ml in mild no perfusion; 338 pg/ml in moderate ischemia, 300 pg/ml or higher in moderate no perfusion; 2570 pg/ml for severe ischemia, and 2000–3000 pg/ml for severe non-perfusion. While in our results, the VEGF value of aqueous humor was found to be 29.29 (13.95, 67.74) pg/ml in mild, 73.85 (72.25, 81.95) pg/ml in moderate, and 90.34 (16.14, 219.11) pg/ml in severe ischemia. In brief, the aqueous humor VEGF value is slightly lower than the vitreous humor VEGF value. Compared with the above standards, the patients in this study were in a state of mild ischemia and mild no perfusion.

Herein, we found that the expression level of IL-6 was positively correlated with the degree of mild, moderate, and severe ME (*P* < 0.001). The reason is that IL-6 can directly induce an elevated expression of VEGF levels and increase the permeability of blood vessels; IL-6 can reduce the expression levels of intercellular connexins and occludins, change the connections between cells, and increase the permeability of blood vessels [6, 8, 17]. A significantly positive correlation has been found between the expression level of IL-6 and the duration of BRVO-ME. The expression level of IL-6 is an important predictor of the transition from ischemia-hypoxia to inflammation. IL-6 is crucial for the transition from acute inflammation to chronic inflammation, linking the inflammatory process and angiogenesis [6, 8, 17, 32–35].

The severity of mild, moderate, and severe ME was strongly linked with ICAM-1 and VCAM-1 (*P* < 0.05). However, the relevant parameters did not differ between the two. ICAM-1 is primarily involved in cell adhesion. By activating VEGFR-2, VEGF increases the expression of ICAM-1 and mediates the adhesion of leukocytes to the retinal vascular endothelium. The more this gene is expressed, the more leukocytes roll and stick to the walls of blood vessels, which can lead to blood stagnation. Simultaneously, abnormal adhesion to vascular endothelial cells and capturing leukocytes can increase the leakage of retinal capillaries and aggravate the degree of edema of ME [6, 8, 13]. VCAM-1 primarily mediates the adhesion between blood vessels. Its upregulation can cause retinal capillary leakage, which in turn leads to the formation of non-perfused areas (NPAs) and neovascularization (NV) [6, 8, 13, 35–37]. Although it has been reported [8, 37–40] that compared with ICAM-1, the increased expression of VCAM-1 is more closely related to the degree of ischemia and hypoxia of the retina and the formation of NPAs and NV. However, the degree of retinal ischemia and hypoxia and the formation of NPAs and NV may result from a variety of factors. We found that both ICAM-1 and VCAM-1 increased with the degree of ME; it was clinically difficult to determine whether ICAM-1 or VCAM-1 was more involved.

## 5. Limitations of the Study

The sample size included in this study was relatively small, and the collected cytokines were few. Furthermore, only the cytokine expression levels of BRVO-ME patients before IRI were analyzed. Thus, more samples are needed and expected for multicentre research.

## 6. Conclusion

VEGF promotes vascular permeability by increasing the phosphorylation of tight junction proteins. This chemokine is used by inflammatory cells to trigger positive feedback loops, aggravating ischemia and hypoxia in the retina, damaging the BRB, and causing leakage, resulting in chronic recurrent ME. IL-6 can be used as a diagnostic or therapeutic target for shifting from ischemia-hypoxia to inflammation and chronicity. Overexpression of ICAM-1, a marker for retinal vascular endothelial cell activation, can increase leukocyte chemotaxis and adhesion, causing leukocyte stasis and even blood flow stagnation, exacerbating obstruction, and widening the obstruction range. Atypical leukocyte adherence to the vascular endothelium can exacerbate retinal leakage in ME. VCAM-1 stimulates leukocyte chemotaxis to the inflammatory site and attaches to the vascular endothelium, damaging it. When overexpressed, it causes retinal vascular endothelial cells to become active. As a result, NPA and NV may arise. In a positive feedback loop, the ME is associated with VEGF, IL-6, ICAM-1, and VCAM-1. Lowering the level of disease-associated cytokines maybe potentially reduce the degree of ME. Therefore, an in-depth study of the levels and the relationship may provide some evidence for the pathogenesis, treatment, and prevention of BRVO-ME.

## Figures and Tables

**Table 1:** Clinical and demographic data.

Index	Numerical value
Age	67.46 ± 8.35 years
Sex (F/M)	27/31
Duration of the disease	15 (9, 15) days
Eye type (R/L)	28/30
Obstruction sites (ST/IT)	38/20
IOP	13.53 ± 2.19 mm·Hg
BCVA (logMAR)	0.5 (0.9, 0.4)
CMT	494 (369, 587) *μ*·m
*Hypertension (Thirty-one/53.45%)*	
SBP	157.36 ± 24.72 mm·Hg
DBP	95.53 ± 18.45 mm·Hg
*Diabetes (twenty-one/34.48%)*	
Baseline HbA1c	7.9 (6.5, 9.6)%
FBS	10.82 ± 1.61 mmol/L
*Hyperlipidemia (eight/13.79%)*	
TC	4.28 ± 0.79 mmol/L
TG	2.12 ± 0.39 mmol/L
HDL-C	1.07 ± 0.17 mmol/L
LDL-C	2.76 ± 0.15 mmol/L

^*∗*^ST, superior temporal; IT, inferior temporal; IOP, intraocular pressure; BCVA, best-corrected visual acuity; logMAR, logarithm of the minimum angle of resolution; CMT, central macular thickness; SBP, Systolic blood pressure; DBP, diastolic blood pressure; HbA1c, glycated haemoglobin; FBS, fasting blood sugar; TC, total cholesterol; TG, triglycerides; HDL-C, high density lipoprotein cholesterol; LDL-C, low density lipoprotein cholesterol.

**Table 2:** BCVA, CTM, and the expression levels of VEGF, IL-6, ICAM-1, and VCAM-1 in the aqueous humor.

Category	Case (n)	BCVA	CMT (*μ*·m)	VEGF (pg/ml)	IL-6 (pg/ml)	ICAM-1 (pg/ml)	VCAM-1 (pg/ml)
Mild	14	0.5 (0.9, 0.4)	338 (277, 349)	29.29 (13.95, 67.74)	3.34 (1.30, 45.59)	64.43 (56.13, 75.37)	128.39 (57.33, 158.54)
Moderate	17	0.5 (0.7, 0.4)	423 (388, 450)	73.85 (72.25, 81.95)	100.40 (31.85, 303.60)	109.57 (92.06, 133.29)	241.45 (211.34, 391.07)
Severe	27	0.7 (1.0, 0.5)	652 (546, 766)	90.34 (16.14, 219.11)	131.12 (10.96, 3914.34)	176.45 (133.29, 190.20)	391.37 (249.01, 525.19)

^*∗*^BCVA, best-corrected visual acuity; CMT, central macular thickness; IL-6, interleukin-6; VEGF, vascular endothelial growth factor; ICAM-1, intercellular adhesion molecule-1; VCAM-1, vascular cell adhesion molecule-1.

**Table 3:** Correlation analysis of BCVA and the severity of ME.

CMT	Mild		Moderate		severe	
BCVA	*r*	*P*	*R*	*P*	*r*	*P*
	0.4575	0.1000	−0.3130	0.2213	−0.0677	0.7371

^*∗*^CMT, central macular thickness; BCVA, best-corrected visual acuity.

**Table 4:** Correlation analysis between BCVA and the expression levels of VEGF, IL-6, ICAM-1, and VCAM-1 in the aqueous humor.

Category	VEGF (pg/ml)		IL-6 (pg/ml)		ICAM-1 (pg/ml)		VCAM-1 (pg/ml)	
*r/P*	*r*	*P*	*r*	*P*	*r*	*P*	*r*	*P*
Mild	0.3379	0.2181	0.1727	0.5382	0.4521	0.0907	0.0361	0.8983
Moderate	0.0470	0.8579	0.4076	0.1044	0.2122	0.4136	0.1781	0.4940
Severe	0.1586	0.4295	0.0593	0.7691	0.0528	0.7936	0.0890	0.6589

^*∗*^BCVA, best-corrected visual acuity; IL-6, interleukin-6; VEGF, vascular endothelial growth factor; ICAM-1, intercellular adhesion molecule-1; VCAM-1, vascular cell adhesion molecule-1.

**Table 5:** Correlation analysis between the severity of ME and the expression levels of VEGF, IL-6, ICAM-1, and VCAM-1 in the aqueous humor.

Category	VEGF (pg/ml)		IL-6 (pg/ml)		ICAM-1 (pg/ml)		VCAM-1 (pg/ml)	
*r/P*	*r*	*P*	*r*	*P*	*r*	*P*	*r*	*P*
Mild	0.9129	<0.0001	0.8159	0.0004	0.8559	<0.0001	0.8931	<0.0001
Moderate	0.9574	<0.0001	0.7751	0.0003	0.5850	0.0108	0.5880	0.0130
Severe	0.8202	<0.0001	0.7438	<0.0001	0.7328	<0.0001	0.4847	0.0104

^*∗*^VEGF, vascular endothelial growth factor; IL-6, interleukin-6; ICAM-1, intercellular adhesion molecule-1; VCAM-1, vascular cell adhesion molecule-1.

## Data Availability

The data used to support the findings of this study are available from the corresponding author upon request.
